# The Influence of Hofmeister Series Ions on Hyaluronan Swelling and Viscosity

**DOI:** 10.3390/molecules13051025

**Published:** 2008-05-01

**Authors:** Aleš Mráček, Júlia Varhaníková, Marián Lehocký, Lenka Gřundělová, Alena Pokopcová, Vladimír Velebný

**Affiliations:** 1 Department of Physics and Materials Engineering, Faculty of Technology, Tomas Bata University in Zlín, Nad Stráněmi 4511, 76005 Zlín, Czech Republic; E-mails: juliavarhanikova@seznam.cz; Grundy.L@seznam.cz; salinka.p@azet.sk; 2 Medical Materials Research Centre, Technology park, University Institute, Tomas Bata University in Zlín, Nad Ovcirnou III 3685, Zlín, 76001 Czech Republic; E-mail: lehocky@post.cz; 3 CPN Ltd., Dolní Dobrouč 401, 561 02 Dolní Dobrouč, Czech Republic; E-mail: velebny@contipro.cz

**Keywords:** Material Science, biomaterials, hyaluronan, Hofmeister series

## Abstract

The dissolution of hyaluronan in water leads to its degradation, and as a result its molecular weight decreases. The degradation of hyaluronan is mainly influenced by temperature, solution composition, and also its pH. This study describes the influence of Hofmeister series ions on hyaluronan behaviour and hyaluronan film swelling by solutions of these ions. It was found that Hofmeister ions show lyotropic effects influencing the entanglement of hyaluronan coils and their expansion from solid polymer films into swollen gel state. The hydrophobic and hydrophilic interactions in the structure of hyaluronan macromolecules are represented by the mutual diffusion coefficient *D*(*c*), the mean mutual diffusion coefficient D_*s*_, the expansion work of coil swelling *RA_δ,s_*, the activation enthalpy of diffusion connected with swelling *H_D,s_* and kinematic viscosity of hyaluronan-ions solutions *ν*.

## Introduction

**Figure 1:**
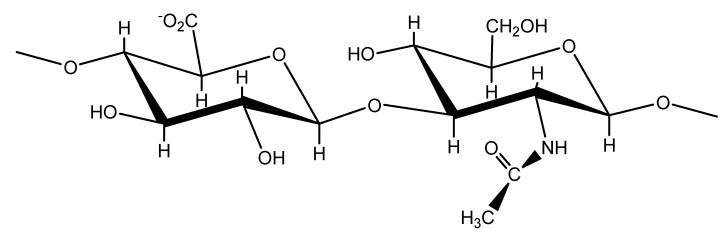
The monomeric unit of hyaluronic acid.

In 1934 Meyer and Palmer described a procedure for isolation of a novel glycosaminoglycan from the vitreous humor of bovine eyes [1]. They showed that this substance contained an uronic acid and an aminosugar, but no sulfoesters. This marked the birth announcement of one of Nature’s most versatile and fascinating macromolecules. It was first isolated as an acid, but it behaved like a salt (sodium hyaluronate) under physiological conditions. Today, this macromolecule is most frequently referred to as hyaluronan, reflecting the fact that it exists *in vivo* as a polyanion and not in the protonated acid form. During the 1930s and 1940s, hyaluronan was isolated from many different sources such as synovial fluids, umbilical cords, vitreous body, skin, rooster comb and also biotechnologically synthesized from streptococci suspensions. Numerous ideas have been offered for applying hyaluronan in different areas and nowadays, many of them are actually used in medicine, pharmacology or in cosmetics. It would take an additional 20 years before Meyer’s laboratory finally completed the work that determined the precise chemical structure of the basic disaccharide unit that forms hyaluronan [2]. During these years they showed that the uronic acid and aminosugar in the disaccharide are D-glucuronic acid and D-*N*-acetylglucosamine, and that they are linked together through alternating *β*–1,4 and *β*–1,3 glycosidic bonds. Both sugars are spatially related to glucose, which in the beta configuration allows all of its bulky groups to be in sterically favourable equatorial positions while all of the small hydrogen atoms occupy the less sterically favourable axial position. Thus, the structure of the disaccharide shown in Figure 1 is energetically very stable [3]. The conformation of hyaluronan acid (HA) has been analysed by a number of techniques showing that the hydrogen bonds are crucial to both secondary and tertiary structures [4].

**Figure 2:**
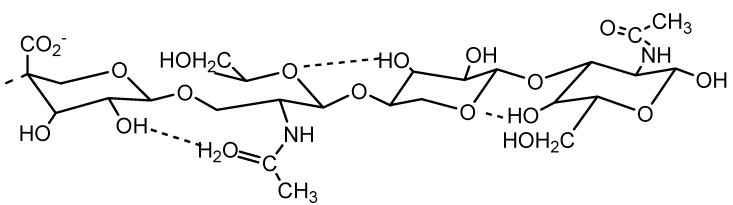
The secondary structure of HA.

The secondary structure of HA (a reducing terminal tetrasaccharide fragment) as a two-fold helix with hydrogen bonds (dotted lines) can be seen in Figure 2. All the indicated H-bonds form in dimethyl sulphoxide solution, whereas in aqueous solution some are replaced by water bridges. In connection with this, it is very useful to study the influence of any ions present in hyaluronan solutions on the secondary and tertiary structure of this polysaccharide.

Ions of the Hofmeister series have a significant effect on the solubility of macromolecular substances and on the stability of their respective secondary, tertiary and quaternary structures. The Hofmeister series (lyotropic series - an order of ability of ions to convert into salt-out or salt-in proteins) are considered to be ions having a typical ion-specific influence on polymers in aqueous systems. The order of Hofmeister anions and cations is:

SCN^- ^< I^- ^< ClO_4_^-^ < NO_3_^- ^< Br^-^ < ClO_3_^-^ < Cl^-^ < BrO_3_^-^ < F^-^ < SO_4_^2-^

K^+^ < Na^+^ << Li^+^ ~ Ca^2+^.

The effects of the Hofmeister series seems to apply mainly in experiments where solvents with higher salt concentrations are used. The initial ions in the series increase the solvent surface tension and decrease the solubility of non-polar "salt-out" molecules, which leads to a strengthening of the hydrophobic interactions. On the other hand, a few final salts in Hofmeister series increase the solubility of non-polar "salt-in" molecules and increase the order in water, which decreases the hydrophobic interactions. These salts interact directly with proteins or polymers and may even be specifically bonded with ions having a strong "salting-in" effect [5, 16,17,18]. Similar ion-effects have been found in the swelling behaviour of several kinds of polymer gels. Suzuki *et al*. [6] investigated the effects of ions on the thermal volume transition in polymer gels and some kind of Hofmeister series effects were detected for the temperature transition. There was discrepancy with the speculations that the origin of ion-effect is caused by the change in water structure around the hydrophobic group [6].

The behaviour of hyaluronan during diffusion process of swelling is presented in this article. The theoretical background behind the computation of mutual diffusion coefficients, mean mutual diffusion coefficients, the expansion work of swelling coils and the activation enthalpy of diffusion connected with swelling H_D,s_ has been fully published by Mracek *et al*. [7].

## Results and Discussion

**Figure 3:**
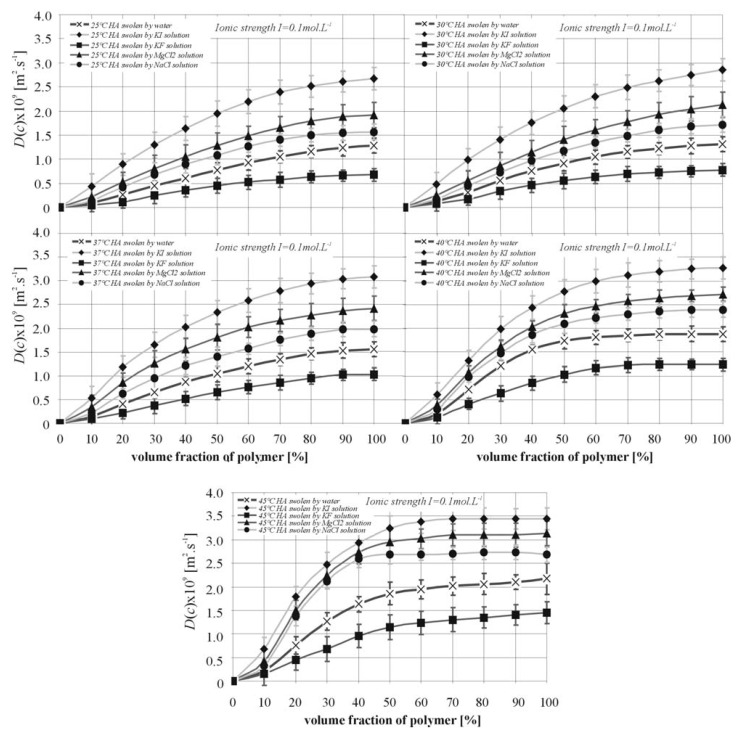
Relation between mutual diffusion coefficient and HA concentration in surface swollen layers.

The influence of Hofmeister series ions on hyaluronan behaviour and hyaluronan films swelling by solutions of these ions can be studied by interferometric methods. The dependence of the mutual diffusion coefficient of swelling on the ability of water to solvate hyaluronan was verified. As can be seen in Figure 3, the relation between the mutual diffusion coefficient and hyaluronan concentration in swollen layer shows a statistically significant decreasing rate of swelling diffusion process for experiment with *KF* addition (Ionic strength *I* = 0.1 mol∙L^-1^) as compared with swelling of hyaluronan by pure water. On the other hand, solutions of *NaCl*, *MgCl_2_* and *KI* force diffusion swelling processes.

**Figure 4:**
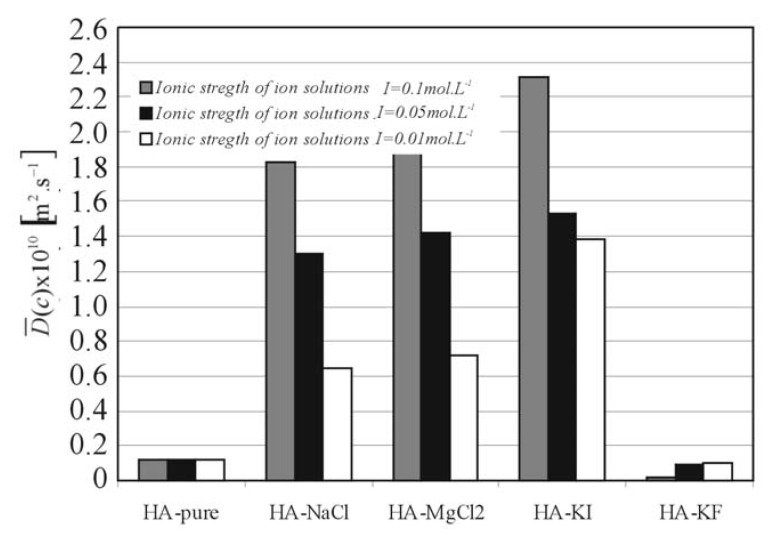
The mean mutual diffusion coefficients of hyaluronan solutions.

Differences between monovalent cations (*Na*^+^, *K*^+^) and divalent cations (*Mg*^2+^) at ionic strength *I* = 0.1 mol∙L^-1^ are consistent with predictions that charge shielding by divalent ions is greater [8, 9]. The data for the swelling diffusion process suggests that *Mg*^2+^ causes greater domain contraction, which is additional to the electrostatically induced changes in HA properties observed with *K*^+^ and *Na*^+^. Individual *Mg*^2+^ ions may co-ordinate two carboxy groups on the same HA chain secondary structure (Figure 2), and promote chain contraction. Consequently, cations of *Mg*^2+^ could induce a significant reduction of chain stiffness. However, the mutual diffusion coefficient *D*(*c*) of swelling shows (Figure 4) nearly order of magnitude differences for *KI* solutions in comparison with *KF* ones. In this case, the influence of the anions (*F*^-^, *Cl*^-^, *I*^-^) on the behaviour of the *K*^+ ^ cation is evident. This fact can be viewed as in accordance with theory of Hofmeister ion series (hydrophilic→ *I*^‑1^<*Cl*^‑1^<*F*^‑1^←hydrophobic). *F*^-^ or *Cl*^-^ ions may alter the co-ordination of water molecules with HA chains, thereby disrupting the hydrogen bonds involved in water bridges. The strong reduction of hydrophilic chain interaction and creation of hairpin loops caused by *F*^-^ can consequently induce a significant increase of chain stiffness. The lower ionic strength shows similar dependences (see in Figure 4), which are represented by the mean mutual diffusion coefficient.

**Figure 5:**
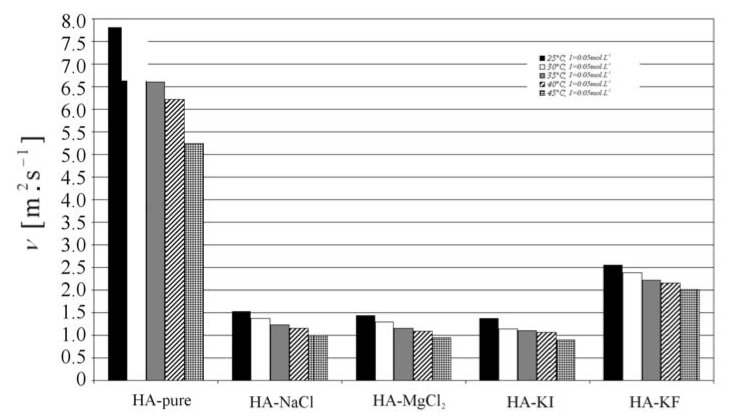
The kinematic viscosity: the comparison of HA-water solutions and HA-ion solutions.

The results and conclusions from the diffusion process of swelling can be compared with results obtained by viscosity measurements (see Figure 5). The additions of ions to aqueous hyaluronan solutions are generally connected with a decrease in the kinematic viscosity. However, the observed higher kinematic viscosity values of *KF*-enriched HA solutions supports the hypothesis of an increase of chain stiffness caused by the presence of *F*^-^.

**Figure 6:**
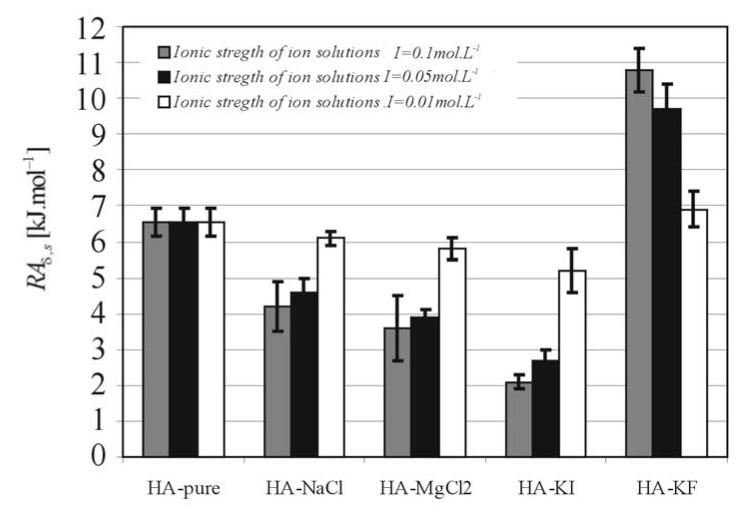
The volume expansion work (*RA_δ,__s_*) resulting from expansion of HA coils during their transition from solid polymer phase into the gel phase of swollen layer.

**Figure 7:**
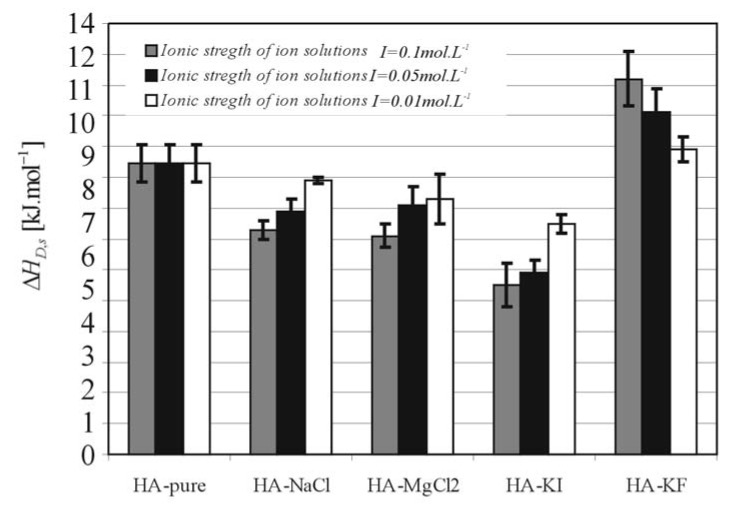
The activation enthalpy of diffusion connected with swelling (Δ*Η_D,s_*) under constant pressure.

Figure 6 and Figure 7 show the relationship between *RA_δ,__s_* and Δ*Η_D,s_*, respectively, and additions of salts (*NaCl*, *MgCl_2_*, *KI*, *KF*) at different ionic strengths (*I* = 0.01 0.05 and 0.1 mol∙L^-1^). These results show that solvation of HA by water is strongly dependent on the ionic strength and particularly on the variety of salt dissociated in the aqueous solutions of HA.

The values of *RA_δ,s_* and *ΔΗ_D,s_* are considerably influenced by the polarity of the basic structural polymer unit - the monomer. Some authors have reported [4, 10,11,12] the important role of hydrogen bonds in hyaluronate chain structures and the associated problem of the relationship between hyaluronate swelling and intermolecular interactions influenced by hydrogen bonds [13,14]. The functionality of hyaluronic acid can mostly be traced back to the swelling properties of polyelectrolyte networks. In aqueous solutions the hyaluronan molecule becomes a negatively charged polyelectrolyte and its size, conformation and its degree of hydration depend on dissociation [13]. Consequently, the affinity between the polymer film and the solvent are very important in the swelling diffusion process, because the solvent molecules must penetrate into the polymer and then the polymer chain molecules must be solvated by solvent molecules. Subsequently, the swelling continues with the expansion of polymer coils and the polymer mobility and permeability increases [15]. As can be seen in Figure 6, the volume expansion work *RA_δ__,s_* for HA-KF is significant higher than for HA-pure and it is evident (see Figure 7), that the values of activation enthalpy of diffusion connected with swelling *ΔH_D,s_* are largely dependent on the variety of salts and the salts on the hydrophobic part of Hofmeister series increase these values. It is assumed that the solvation of HA with ion solutions on the hydrophobic part of Hofmeister series results in the decreasing flexibility of the macromolecular coil and results in an increase of the potential barrier which must be overcome during the diffusion process of swelling.

The results obtained support the hypothesis of hydrophilic-hydrophobic interaction [19] in hyaluronan chains swollen by Hofmeister ions solutions. This hypothesis was verified by diffusion process of swelling and viscosity measurements. With respect to applications of hyaluronan in ophthalmology, surgery and wound healing, it will be very interesting to study the behaviour of hyaluronan in the water solutions with additions of quaternary ions (for example hexadecyltrimethyl-ammonium bromide). It is known that these quaternary ions create hyaluronan precipitates in aqueous solution. The results (the influence Hofmeister ions on swelling and kinematic viscosity of hyaluronan solutions) reported in this paper can be used for re-conversions of these precipitates. Accordingly, our next results concerning to this hyaluronan precipitates problem will be published shortly.

## Experimental

### Materials

The hyaluronan samples (rank 150806-D1) were obtained from Contipro, Ltd., Dolní Dobrouč, Czech Republic, (molar mass *M*_w _= 630x10^3 ^g∙mol^-1^). All materials and chemicals used for the experiments were of standard purity p.a. The following salts in solutions (redistilled water + salt) for swelling process and viscosity measurements were used: sodium chloride, *NaCl* (Fluka); magnesium chloride, *MgCl*_2_ (Fluka); potassium fluoride, *KF* (Fluka); potassium iodide, *KI* (Sigma-Adrich). The process of swollen surface layer formation was studied on the solid films of polymer samples prepared by casting from the 0.01M concentration solutions in redistilled water and by subsequent evaporation in a desiccator.

### Experimental setup and measurments

**Figure 8:**
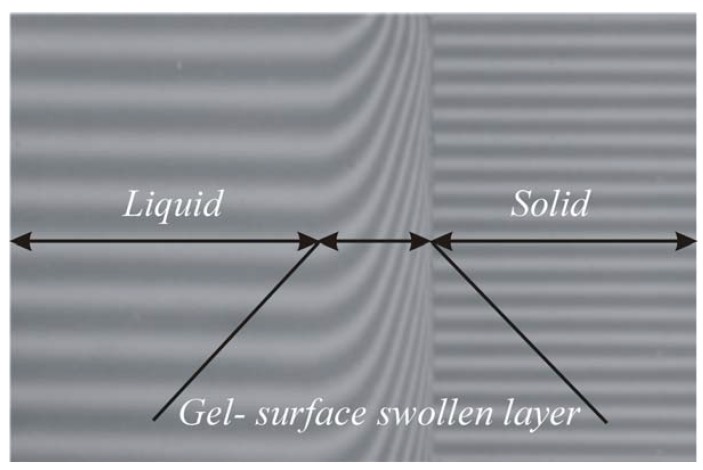
A typical interferogram obtained from swelling diffusion process measurement.

The swelling diffusion process measurements were done using the previously described wedge interferometer [7, 8]. Experiments were performed at five temperatures (25, 30, 37, 40 and 45°C). The mutual diffusion coefficient *D*(*c*) and mean mutual diffusion coefficient of swelling (D_*s*_) at definite times were measured and computed from recorded interferograms. A typical interferogram can be seen in Figure 8, which shows the concentration field of hyaluronan on the surface swollen layer expressed as the change of refractive index.

Samples of thin hyaluronan films were put in a temperature-controlled holder of (23x20x3.5)x10^-9 ^m^3 ^volume between two semi-transparent glass plates with a transparency of about 40%. The temperature was controlled exactly constant to a deviation of ±0.1°C. In each measurement, 3-5 shots were taken at regular intervals. The final resultant interferograms were scanned with a NIKON COOLPIX 4500 digital camera. The SAIA software developed by Urban and Mracek (Department of Physics and Materials Engineering, FT, TBU in Zlin) was used for the image analyses of the interferograms. The factors *RA_δ,s_*, *ΔH_D,s_*, were calculated using logarithmic form formulas [7, 8], consequently, standard estimatation errors for *RA_δ,s_* and *ΔH_D,s_* were computed for confidence interval at 80% significance level. The kinematic viscosity *ν* values were obtained from measurements executed on an Ubbelohde viscometer (*C* = 0.01) at five temperatures (25, 30, 37, 40 and 45°C) and the ionic strength of solvents was *I* = 0.05 mol∙L^-1^.
